# Stability testing of the Pfizer-BioNTech BNT162b2 COVID-19 vaccine: a translational study in UK vaccination centres

**DOI:** 10.1136/bmjos-2021-100203

**Published:** 2021-09-12

**Authors:** Laila Kudsiova, Alison Lansley, Greg Scutt, Marcus Allen, Lucas Bowler, Sian Williams, Samantha Lippett, Selma Stafford, Michael Tarzi, Michael Cross, Michael Okorie

**Affiliations:** 1 Centre for Stress and Age-Related Disease, and Centre for Regenerative Medicine and Devices, School of Applied Sciences, University of Brighton, Brighton, UK; 2 Centre for Stress and Age-Related Disease and Biomaterials and Drug Delivery Research and Enterprise Group, School of Applied Sciences, University of Brighton, Brighton, UK; 3 Medicines Optimisation Research and Enterprise Group, School of Applied Sciences, University of Brighton, Brighton, UK; 4 Centre for Stress and Age-Related Disease, School of Applied Sciences, University of Brighton, Brighton, UK; 5 University Hospitals Sussex NHS Foundation Trust, Brighton, UK; 6 Independent General Practitioner, Brighton, UK; 7 Brighton and Sussex Medical School, Brighton, UK

**Keywords:** COVID-19, vaccine, spike protein, RNA, messenger, lipid nanoparticles

## Abstract

**Objective:**

The roll-out of the Pfizer-BioNTech BNT162b2 COVID-19 vaccine has brought many logistical challenges, such as the absence of comprehensive stability data leading to strict handling instructions during dilution and administration. Accidental mishandling therefore presents challenging clinical dilemmas, which often led vaccine providers to err on the side of caution and discard mishandled vials rather than risk administering ineffective vaccine. This study aims to answer key questions about the vaccine’s stability to allow for a more informed decision-making process should a non-conformity occur.

**Methods:**

Residual vaccine in freshly used, but appropriately stored vials collected from vaccination centres in Brighton, UK, were tested after exposure to various handling conditions and analysed by dynamic light scattering to determine the size of the lipid-mRNA nanoparticles, and gel electrophoresis to visualise the mRNA integrity and separation from the lipid formulation.

**Results:**

Knocking or dropping vaccine samples from small heights resulted in lowest levels of instability, indicating low risk of compromising clinical efficacy. However, repeated drawing and injecting through 23 G needles at high speed and, more significantly, shaking and vortexing led to progressive increase in the size and polydispersity index of the lipid-mRNA nanoparticles, coupled with or caused by up to ~50% release of mRNA from the lipid formulation. This is thought to impact the vaccine’s efficacy due to lack of free mRNA protection and cellular internalisation.

**Conclusions:**

These results reiterate the importance of adhering to the manufacturer’s instructions on handling, especially with regard to shaking and exposing the vaccine to excessive vibration.

**Strengths and limitations of this study**

- This translational study answers key questions raised in COVID-19 vaccination centres regarding the stability of the Pfizer-BioNTech COVID-19 vaccine on handling, to inform decision-making process should accidental mishandling or non-conformity occur.
- The close collaboration between healthcare professionals in vaccination centres and scientists at the University of Brighton allowed for timely access to residual vaccine, accurate data collection and testing of handling conditions highly relevant to clinical practice.
- The validity of the results was confirmed through repeated testing on at least three separate occasions.
- Since vaccine vials were prioritised solely for vaccination use during the peak of the pandemic, tests were performed on small samples aliquoted into plastic tubes (up to 0.3 mL, equivalent to one vaccine dose) taken from residual vaccine in used but appropriately handled bottles, rather than full six-dose glass vials.
- The detected instability upon mishandling indicates a reduction in the vaccine’s efficacy; however, clinical activity was not directly measured in this study.

## Introduction

The regulatory approval of COVID-19 vaccines, including the BNT162b2 COVID-19 mRNA (Pfizer-BioNTech) vaccine, has offered the world a ray of hope to accelerate the end of the COVID-19 pandemic. However, the huge vaccine roll-out of the Pfizer-BioNTech vaccine in the UK has not gone without its challenges, mainly due to the logistics involved in its distribution and storage at very low temperatures. Other factors also posed significant hurdles to its use; for instance, the strict handling instructions which were specified as part of the conditions of authorisation resulted in the need to recruit more healthcare professionals (or trained healthcare students) to help with the dilution, drawing up and administration of the vaccine. This has also caused a degree of uncertainty about the potential adverse consequences of accidental mishandling of the vaccine. Pfizer’s guidelines currently state that the vaccine vials should only be inverted gently 10 times after thawing and dilution with saline, and should not be shaken.^1 2^ Further instructions stated that diluted vaccines should not be transported by motor vehicles away from the site of dilution, due to possible exposure to vibrations,^3^ suggesting that any vibrational stress may destabilise the vaccine or reduce its efficacy. Although a follow-up study conducted by Pfizer to mimic exposure to vibration showed that diluted vaccine maintained its measured quality attributes during ground transportation,^4^ no data have been provided about the effect of moderate to vigorous shaking of diluted vaccine, the effect of repeated or rapid drawing of the vaccine through needles, or the accidental dropping of vials or syringes, which particularly led many vaccine providers to err on the side of caution and discard precious vaccine. Without this information, it is not known whether viable vaccine is wasted unnecessarily or whether extra precautions should indeed be taken to minimise loss of efficacy due to mishandling.

A successful multidisciplinary collaboration, initiated at the start of the UK’s vaccine roll-out, comprising a psychologist, pharmacists, a clinical pharmacologist, nurses, scientists, immunologists and medical staff from University Hospitals Sussex NHS Foundation Trust, Brighton and Sussex Medical School, the Primary Care Network, Sussex Community Trust and the University of Brighton, allowed for stability testing of the Pfizer-BioNTech vaccine to answer those key questions. Residual vaccine from freshly used but appropriately stored vials was tested for its physical stability using two techniques to determine (1) signs of aggregation, by measuring the size of the vaccine’s mRNA-encapsulating lipid nanoparticles by dynamic light scattering, and (2) signs of mRNA degradation or release from the lipid nanoparticles by agarose gel electrophoresis. Three different types of handling conditions were tested to mimic clinical practice: determining the effect of shaking and exposure to vibrations, dropping the vials from various heights and drawing through a 23 G needle repeatedly and at increasing speeds. This article therefore provides further details on the consequences of vaccine mishandling, to inform the decision-making process regarding the integrity of the Pfizer-BioNTech vaccine across vaccination centres in the UK and worldwide.

## Materials and methods

### COVID-19 vaccine handling

Used but freshly diluted vials (from batches EL7834 Exp 04/2021, EL8713 Exp 05/2021 and EN1185 Exp 05/2021) containing any residual volume of the COVID-19 Pfizer-BioNTech vaccine after five or six doses had been dispensed were obtained from the vaccination centres at the Brighton Racecourse and the Royal Sussex County Hospital in Brighton, UK. The vials had been stored under recommended conditions and tested within 6 hours of dilution. Apart from the necessary ~10 min car journey to the university campus laboratory, the vials were not subjected to any additional vibrations. All equipment (pipette tips and Eppendorf tubes) used in the handling of the vaccine were certified DNase/RNase-free and glassware was rinsed copiously with DNase/RNase-free water (Merck Chemicals, Notts, UK) before use. Gloves and a face mask were worn throughout the duration of the experiments to avoid mRNA degradation due to naturally present RNases on the surface of the skin. DNase/RNase-free water was also used to reconstitute buffers used in electrophoresis experiments. Every experiment was repeated on at least three separate occasions to ensure that the results were reproducible.

Aliquots (60 or 300 µL) from pooled volumes of 4–10 vaccine vials (which were pretested to check if they were all of similar particle size) were gently pipetted into 1.5 mL capacity DNase/RNase-free Eppendorf tubes (ThermoFisher Scientific, Staffordshire, UK), which were then subjected to three main types of handling conditions to mimic different clinical scenarios: (1) samples were shaken to varying degrees; by gentle inversion (the current recommended practice), flicking or shaking at moderate speed by moving up and down 10 times (similar to shaking applied when reconstituting injections), shaking vigorously (similar to shaking an oral suspension), or subject to vibration by vortexing on a vortex mixer (Fisher Scientific, UK) set at a maximum speed for 10 s; (2) samples were knocked over on a bench or dropped from various heights (10, 30 or 130 cm) onto a bench or laminate flooring; and (3) samples were drawn and injected through a 23 G needle up to five times, at different speeds (up to 300 µL/s). Controls were used in every experiment and were left untreated.

### Dynamic light scattering

Approximately ~45 µL from each of the above handled samples were transferred to disposable low volume (45 µL) polystyrene clear cuvettes (purchased from Malvern Panalytical, Worcestershire, UK) without further dilution, and the apparent hydrodynamic size of the vaccine formulation was measured by dynamic light scattering at 25°C using a Zetasizer Nano ZS (Malvern Panalytical) as described previously.^5 6^ Three repeat measurements (of 13–17 runs each) were performed for each sample. Results were statistically analysed by analysis of variance (ANOVA) followed by a Dunnett post-test.

### Gel electrophoresis

Agarose gel electrophoresis was performed to visualise mRNA. Migration of the mRNA band can reveal the type of instability that the various handling conditions might inflict on the mRNA within the lipid formulation. Ten microlitres from each of the above samples was transferred into separate DNase/RNase-free Eppendorf tubes without further dilution, and 2 µL of gel loading buffer (containing 0.25% w/v bromophenol blue and 40% w/v sucrose, purchased from Merck Chemicals) was added to each sample. A 10 µL volume of the final reconstituted vaccine was estimated to contain ~1 µg of encapsulated mRNA; however, not all the mRNA dose was expected to be visible on the gel since only free (uncondensed or unencapsulated) mRNA was expected to migrate down the gel. To release the full dose of mRNA, for comparison purposes, unhandled 10 µL samples were treated with 2 µL of 0.1% v/v Triton X-100 (Merck Chemicals) to disrupt the lipid nanoparticles and release the mRNA. An RNA ladder (500–9000 bases; New England BioLabs, UK) was also used as a reference to verify the size of the mRNA in the vaccine.

Control or test samples were loaded onto a 1% w/v agarose (Fisher Scientific) gel in tris–acetate–EDTA buffer (pH 8.3) prepared in DNase/RNase-free water containing 2 µL of GelRed nucleic acid stain (Cambridge Biosciences, UK) as described previously.^5 7^ The gel was run at 100 V for 30 min and then visualised under UV light using an AlphaImager (Alpha Innotech, UK).

## Results

### Effect of shaking and vortexing

**Figure 1:**
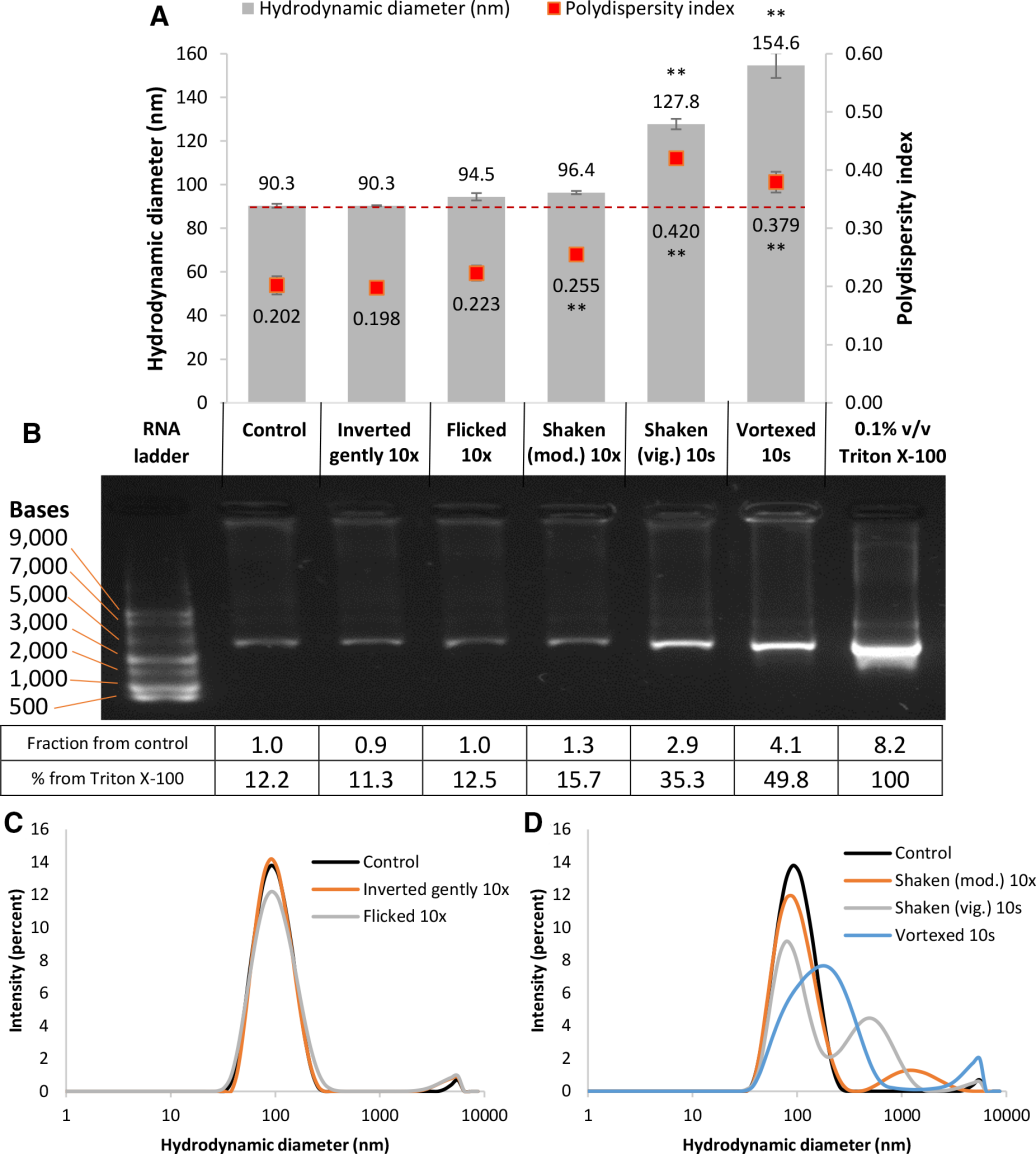
(A) Hydrodynamic diameter (left axis) and polydispersity index (right axis) of COVID-19 Pfizer/BioNTech vaccine nanoparticles upon subjecting the vaccine to gentle inversion, flicking, shaking mod., shaking vig. or vortexing. Numbers above bars are hydrodynamic diameter values (nm). Numbers below the scatter points are polydispersity indices. **Significant difference when compared to the control (analysis of variance with Dunnett post-test, p<0.01). Each data point was an average of three measurements±SD. (B) Gel electrophoresis of corresponding samples labelled from the graph above showing mRNA release from the lipid formulation. The first band in the gel is an RNA ladder, and the last band is vaccine treated with 0.1% v/v Triton X-100, representing 100% mRNA release from the formulation. The table under the gel is a semiquantification of the band intensity using ImageJ analysis, showing fractional mRNA increase in intensity compared to the control and the % release compared to a sample treated with Triton X-100. (C, D) Average size distribution plots by intensity of the corresponding samples (A). mod., moderately; vig., vigorously.

The effect of shaking and vortexing was tested in order to mimic handling conditions during dilution and mixing with saline in the clinical setting, and to simulate more extreme levels of shaking or exposure to vibration. The size measurement and gel electrophoresis results are shown in figure 1. In comparison to the untreated control, no significant difference was observed in the lipid particle size or polydispersity index (a measure of heterogeneity or variability in size distribution) when the sample was inverted gently or flicked 10 times (although a slight increase in average size and polydispersity index was observed in the latter sample as shown in figure 1A, despite not being significantly different). Particle size grew significantly (p<0.01; ANOVA, Dunnett post-test) in vigorously shaken and vortexed samples. The polydispersity index also increased significantly (p<0.01) in samples that were moderately shaken, vigorously shaken and vortexed. It should be noted that despite only a slight increase in the average particle size, for example, a 6.1 nm increase in the case of the moderately shaken sample compared to the control, the coinciding increase in polydispersity index (from 0.202 to 0.255) indicated the presence of a small number of much larger particles, the presence of which increased the average population size. This was evident from the intensity size distribution plots (shown in figure 1C, D), which showed a reduction in the intensity of the main size peak and either the appearance of another larger size distribution peak or a widening in the distribution of the main peak towards the larger particle size range, as opposed to the lower size range, suggesting that the polydispersity index increase was due to the presence of larger and not smaller particles. In the case of the moderately shaken sample (orange line in figure 1D), the larger-sized population peaked at around 1000 nm, suggesting the presence of a small number of very large particles. In the case of the vigorously shaken and vortexed samples, larger second peaks or broadening and shifting of the original size peak suggested the presence of an even higher proportion of larger particles, indicating a higher degree of aggregation.

Gel electrophoresis (figure 1B) shows the migration of free (unencapsulated) mRNA down the gel and the position of the band reflects its molecular weight. In comparison to a reference RNA ladder, the size of the vaccine’s mRNA appears to be between 3000 and 5000 bases long, which matches the expected size of the modified spike mRNA protein in the BNT162b2 vaccine, reported to be 4284 bases long.^8^ The samples tested (which were the same as those used for size measurement) showed a corresponding increase in the mRNA band intensity with increasing levels of shaking and vortexing, suggesting that more free mRNA has been released from the lipid formulation and was available to travel down the gel. The increased gel band intensity highly correlated with the increase in particle size (R^2^ value was measured to be 0.9954), suggesting that the observed aggregation was related to the mRNA release from the lipid nanoparticle formulation. It should be noted, however, that no degradation or breakdown of the size of the mRNA was observed due to the absence of any smaller molecular weight bands or smears underneath the main mRNA band in the tested samples. This is thought to be attributed to modifications made to the mRNA, to improve its stability to enzymatic degradation.^8^

In order to get an idea of the approximate amount of mRNA separation, vaccine samples were treated with 0.1% v/v Triton X-100 to release the encapsulated mRNA. Band intensity analysis using ImageJ (which provides an approximate semi-quantitative estimate of band brightness) is shown in the table within figure 1B, suggesting that the amount of mRNA release due to Triton X-100 was around 8.2-fold higher compared with control (which equated to ~12.2% of mRNA release from the control relative to that released using Triton X-100). This also equated to ~2.9-fold and 4.1-fold increases in mRNA release in the vigorously shaken and vortexed samples, respectively, when compared with the control. This was also estimated to be ~35% and~50% of the overall mRNA dose, respectively.

### Effect of dropping from various heights

**Figure 2:**
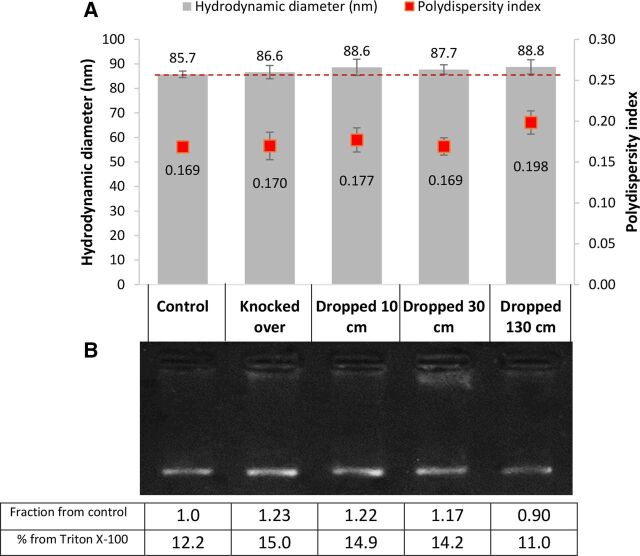
(A) Hydrodynamic diameter (left axis) and polydispersity index (right axis) of COVID-19 Pfizer/BioNTech vaccine nanoparticles upon knocking over or dropping from various heights. Numbers above bars are hydrodynamic diameter values (nm). Numbers below the scatter points are polydispersity indices. Results were not significantly different from the control (analysis of variance with Dunnett post-test). Each data point was an average of three measurements±SD. (B) Gel electrophoresis of corresponding samples labelled from the graph above showing mRNA release from the lipid formulation. The table under the gel shows semiquantification of the band intensity using ImageJ, showing fractional mRNA increase in intensity compared to the untreated control and the % release compared to a sample treated with Triton X-100.

Vaccine samples were knocked over on a bench or dropped from various heights to mimic accidental dropping during transportation, dilution or administration. Size measurement results (figure 2A) showed no significant difference in the actual size or the polydispersity index when compared with the control. However, a slight increase in size was observed in all samples, which was highest in the sample dropped from a 130 cm height (an average increase of 3.1 nm) mirrored by a slight increase in polydispersity index, suggesting minor aggregation. The size intensity distribution of the samples was also comparable to that of the control (data not shown). The release of mRNA from the lipid formulation was also similar to that of the control, as shown from gel electrophoresis results in figure 2B. Some fluorescence was detected just underneath the well, which could be due to partial mRNA release from the complex that was not fully released to travel down the gel, but remained loosely associated with the lipid nanoparticles. This was mostly evident in samples dropped from a 30 cm height.

It should be noted that 60 µL sample volumes were used in the experiments used for figures 1 and 2; however, larger samples of 300 µL were also tested in selected samples and showed very similar results (online supplemental figure S1 in online data repository).

### Effect of repeated drawing and injection through a 23 G needle at different speeds

**Figure 3:**
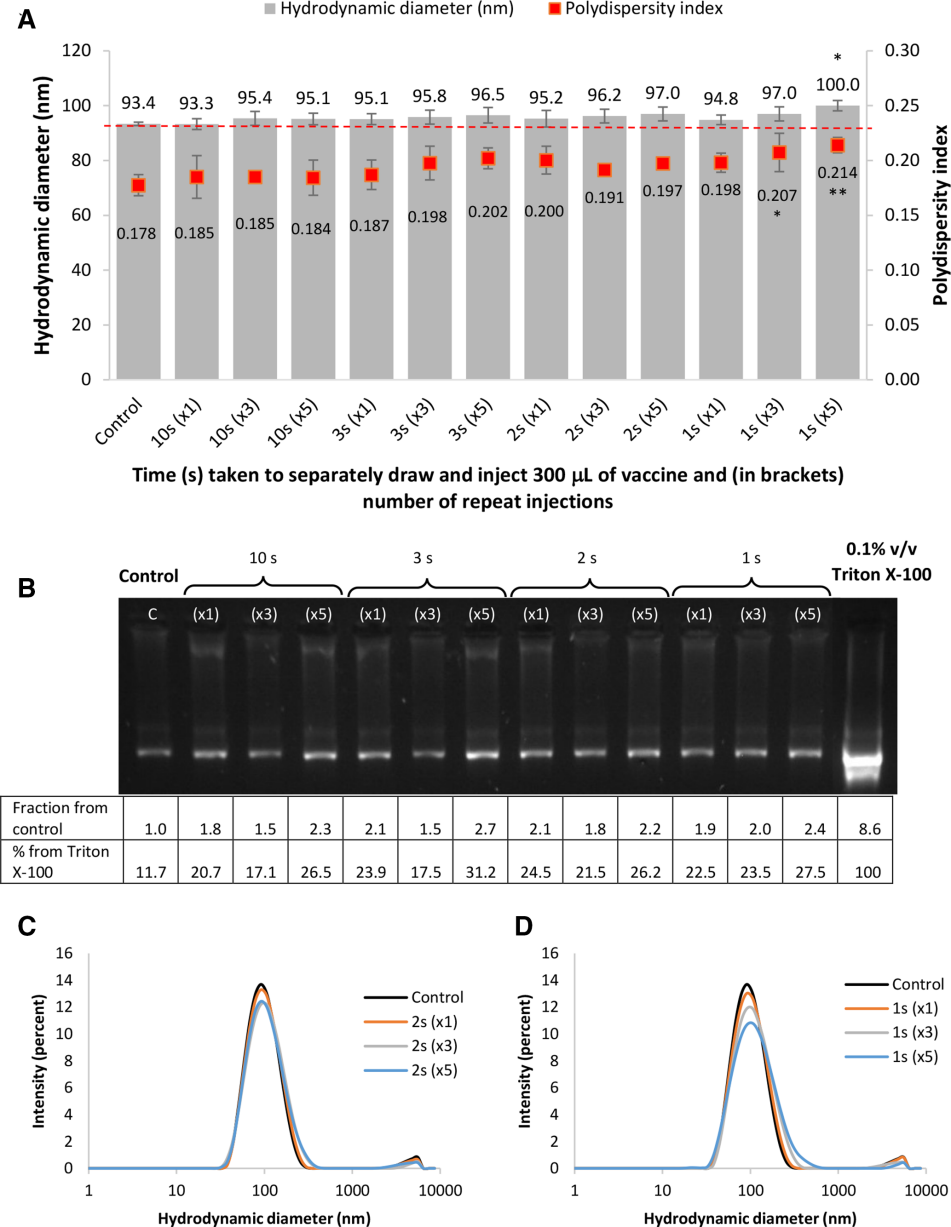
(A) Hydrodynamic diameter (left axis) and polydispersity index (right axis) of COVID-19 Pfizer/ BioNTech vaccine nanoparticles upon injecting a 300 μL dose through a 23 G needle over 10, 3, 2 and 1 s, once, three or five times. Numbers above bars are hydrodynamic diameter values (nm). Numbers below the scatter points are polydispersity indices. * and ** indicate a significant difference when compared to the control (analysis of variance with Dunnett post-test, p<0.05 and p<0.01, respectively. Each data point was an average of three measurements±SD. (B) Gel electrophoresis of corresponding samples in the graph above showing mRNA release from the lipid formulation. The last band in the gel is vaccine treated with 0.1% v/v Triton X-100, representing 100% mRNA release from the formulation. The table under the gel is a semiquantification of the band intensity using ImageJ, showing fractional mRNA increase in intensity compared to the control and the % release compared to a sample treated with Triton X-100. (C, D) Average size distribution plots by intensity of 2 s and 1 s injection times of the corresponding samples (A).

Each Pfizer/BioNtech multidose vaccine contains between five and six doses, which means that during administration, some of the vaccine volume may be drawn up and expelled through a needle multiple times (e.g. to adjust the volume in the syringe or expel bubbles). Furthermore, the speed at which vaccine is drawn and expelled through a needle may also affect the integrity of the lipid nanoparticles. To assess whether encapsulated mRNA vaccine is damaged during this process, 300 µL volume (equivalent to one dose) of vaccine was drawn and released through a 23 G needle (the recommended needle size) once, three or five times, and at different speeds, to mimic the number of times doses are removed and variation in speed of injection in clinical practice. Drawing and releasing the syringe content was counted as one injection cycle. Sizing results in figure 3A showed very small, mainly insignificant differences. However, particle size generally grew progressively with increasing number of injections, as well as the speed of injection. Although a small increase in particle size and polydispersity index was observed in most samples, a significantly larger particle size was detected only in the sample injected very quickly (delivering the whole 300 µL dose in one second) and repeated five times. With regard to polydispersity index, a significant difference was observed in the 1 s injection (repeated both three and five times). This is reflected by a decrease in intensity size peak, and a slight widening and rightward shift in the intensity size in figure 3D. This observation indicates an increase in particle size and a larger distribution of particle sizes.

Gel electrophoresis results (figure 3B) showed a slight increase in band intensity, indicating further mRNA release from the formulation; however, it was most pronounced with increasing the number of injections (ie, five injections), in which case up to 2.7-fold increase in mRNA release was detected compared with the control (this equated to ~31.2% release compared with Triton X-100 treatment).

## Discussion

This study highlights details that are, to our knowledge, not yet available in the literature, regarding the physical stability of the Pfizer-BioNTech COVID-19 mRNA BNT162b2 vaccine. These results are particularly pertinent in view of the special precautions which have to be observed in handling this first in class mRNA vaccine.

### Physical stability

Due to the prioritised vaccine’s clinical use during the peak of the pandemic, it was deemed unethical to test (and therefore waste) full multidose bottles of vaccine, which is why samples from remaining vaccines after the doses were removed were tested in smaller volumes (up to 300 µL) in plastic tubes, which were comparable to single vaccine doses in plastic syringes. Interestingly, the results showed that the vaccine’s stability can be compromised when mishandled, mainly by shaking the vaccine or exposing it to vibrations, and to a lesser extent, on repeated injection through a 23 G needle at high speed (300 µL/s). Knocking or dropping the vaccine from various heights showed minor changes, however they were not significantly different from the control. The physical instability of the vaccine appears to mainly stem from particle aggregation, coupled with or caused by the release or separation of the mRNA from the lipid formulation. This is strongly evident in the case of shaken and vortexed samples (figure 1) where an extremely high correlation between particle size and the amount of mRNA release was observed (R^2^ value=0.9954).

Due to practical reasons, and to ensure that the vaccine was collected and tested within 6 hours of dilution, different vaccine batches were tested on separate occasions. It was noted that the size and polydispersity index of the untreated controls varied slightly between batches, as shown in figures 1–3. This is most probably due to batch-to-batch variability, or slight differences in handling conditions before obtaining the vials. However, reassuringly, repeat experiments performed on batches with slightly different starting size showed a very similar and proportional increase in particle size and polydispersity index, as well as mRNA release when tested under the same conditions. Notably, untreated control samples consistently showed around 12% of free mRNA migration down the gel, which was detected in every gel electrophoresis experiment. This could be attributed to the incomplete encapsulation efficiency of the mRNA (estimated from these experiments to be around 88%), which may be deemed within expected and acceptable limits by the manufacturer. Comparably, encapsulation efficiency of similar lipid nanoparticle formulations, also containing the mRNA spike protein, was reported to be around 92%.^9^

### Vaccine efficacy

Although the biological activity of the vaccine’s mRNA expression was not tested in this study, it is well recognised that naked mRNA that is not complexed or encapsulated within its delivery vector is highly unstable in the extracellular environment due to degradation by RNases.^10–12^ Although the mRNA used in the Pfizer COVID-19 vaccine has been modified to minimise enzymatic degradation^8^ (which was consistent with the gel electrophoresis results conducted in this study due to the absence of degradation bands), the fact that a higher proportion of the mRNA dose was released from its delivery vector was still concerning. In the absence of a delivery vector, free mRNA does not have the ability to diffuse across cell membranes due to its large molecular weight (estimated to be ~1.5 MDa) and its anionic charge, which is repelled by the negative charges of the proteoglycan-coated cell membrane.^10 13^ Since mRNA requires cellular machinery to synthesise and express the viral spike protein that generates the immune response, the vaccine’s efficacy is expected to be reduced if the mRNA is released from its formulation. Based on the results obtained, it is therefore highly probable that the vaccine loses some of its efficacy when mishandled.

Another implication of the observed instability could be attributed to the aggregation of the vaccine formulation, since large particles may not be internalised into cells by endocytosis as efficiently as smaller particles, leading to a reduction in the vaccine’s efficacy. A study conducted by Moderna Therapeutics^14^ found that lipid nanoparticles encapsulating mRNA vaccine (similar in formulation to the Pfizer-BioNTech vaccine) showed best efficacy when the particle size was between 75 nm and 95 nm in diameter, both in terms of mRNA expression and immunogenicity. Larger (up to 140 nm) or smaller (down to 50 nm) particles resulted in reduced efficacy.^14^ Furthermore, nanoparticles in the size range 10–100 nm, injected via the intramuscular route, can enter the lymphatic system readily and drain to lymphoid organs rapidly (within hours of injection), which is vital for the vaccine’s efficacy since that permits direct access to dendritic cells and uptake and presentation to B cells.^15 16^ Nanoparticles larger than ~200 to 500 nm do not enter the lymph nodes as efficiently and take over 24 hours to drain into lymph nodes, thereby reducing the efficacy and stability of the mRNA in vivo.^17^

An important observation worth noting is that the instability stemming from both aggregation and mRNA release is progressive in nature, depending on the amount of force applied during handling, and significant instability was detected even though small sample volumes and plastic tubes were used. In figure 1, vigorous shaking resulted in higher instability compared with moderate shaking or flicking, and in figure 3, repeated and quicker drawing also showed increase in size, polydispersity index and mRNA release. Minimising the number of handling steps involved between dilution and administration to patients is therefore desired while reiterating the importance of adhering to manufacturer’s handling instructions.

## Conclusions

The results of this study indicate that handling of the Pfizer-BioNTech COVID-19 mRNA BNT162b2 vaccine requires care and attention, as specified by the manufacturers. However, further detail is provided on potential breaches in handling which might impact the efficacy of the vaccine in a clinical setting. Repeated and rapid drawing up and injection of the vaccine doses should be minimised. Knocking over or dropping vaccine samples from small heights showed a low level of physical instability, indicating low risk of compromising efficacy; however, it should be noted that small volume samples in plastic tubes rather than full multidose glass vials were tested, and biological efficacy has not been directly assessed in this study.

The compromise in physical stability seen, particularly with shaking or exposing the vaccine to vibrations, endorses the need for close supervision of vaccine preparation, handling and administration in order to maintain standards. As the experience of vaccinators increases, adherence to pharmacy-approved standard operating procedures continues to be an important control in reducing the risk of vaccine degradation.

The desire to minimise wastage balanced against the risk of administering ineffective vaccine can create conflict in clinical decision making, and our data provide some indication and reassurance of vaccine stability following very minor breaches in clinical use.

## Data Availability

Data are available in a public, open access repository. Raw data and analysis are available in repository entitled: Data for 'Stability testing of the Pfizer-BioNTech BNT162b2 COVID-19 vaccine – a translational study in UK vaccination centres' at the following URL: https://researchdata.brighton.ac.uk/id/eprint/271.
